# Effect of Shear History on Solid–Liquid Transition of Particulate Gel Fuels

**DOI:** 10.3390/gels9110902

**Published:** 2023-11-15

**Authors:** Jian Li, Yaning Li, Wei Xiao, Jingyan Wang, Boliang Wang

**Affiliations:** 1 School of Chemistry and Chemical Engineering, Nanjing University of Science and Technology, Nanjing 210094, China; lijian@njust.edu.cn (J.L.); lyn_00446@163.com (Y.L.); wangjingyan2020@126.com (J.W.); 2 Chongqing Hongyu Precision Industrial Group Co., Ltd., Chongqing 402760, China; iridescent_bubbles@163.com

**Keywords:** gel fuels, microstructure, solid–liquid transition, thixotropy, local shear, shear history

## Abstract

Investigating the structural evolution of particulate gels is a very challenging task due to their vulnerability and true flow characteristics. In this work, deeper insight into the rheological properties of gel fuels filled with fumed silica (FS) and aluminum microparticles (Al MPs) was gained by changing shear procedures. Firstly, the flow curves were found to no longer follow the monotonic power law and exhibited subtle thixotropic responses. As the shear rate increased, the gel structure underwent a transition from local shear to bulk shear in the nonlinear region after yielding. This finding reveals the prevalence of nonideal local shear in industry. Secondly, the time-dependent rheological responses demonstrated that the strength spectrum of gel fuels depends on the applied shear rate, with stress relaxation more easily observed at lower shear rates. Those results involved the structural disruption, recovery, and equilibrium of particulate gels from two scales of shear rate and shear time.

## 1. Introduction

Gel fuels filled with composite particles possess the enhanced rheo-physical property and better performance of combustion and explosion [1,2,3], such as higher combustion rates [4,5] and catalytic decomposition [6,7]. These benefits allow gel fuels to be used for a wider range of advanced propulsion systems. However, the parameters of processing, transportation, or atomization are determined by the non-Newtonian fluid characteristics, which are related to variations of the particulate anisotropic structures [8]. Particulate gel will possess yield stress or thixotropy in the most basic scenarios where only hydrodynamic and contact interactions exist [9]. In fact, solid–liquid transition is tightly associated with particulate flocculation [10], preparation conditions [11], and aging effects [12], and determines the actual gelation, as well as the subsequent performance in handling. However, the importance of shear history is increased, since time scales associated with the dynamics of thixotropy or aging in structured gels are longer than any viscoelastic one [13].

As the basis for functionalized fuel design, FS has become a commonly adopted gelation scheme for its ease of acquisition and exhaustive applicability. Entangled clusters tend to rearrange in response to mechanical [14] or acoustic [15] stimuli. Because of weak hydrogen bonds, subtle competitions between shear dissociation and bonding interaction occur [16]. With an increasing shear rate, clusters gradually collapse and exhibit wall slip, two-step-yielding, and shear-induced migration [10,17]. As a result, understanding shear history is a prerequisite for the in-depth study of the structural characteristics of particulate dispersions.

Pre-shear is a common method for eliminating shear history. However, there is no uniform criterion to judge the efficacy of existing protocols. Typically, an ideal protocol would include (but not be limited to): (1) avoiding improper operations that mask the original signal, such as preshearing or unsuitable test methods such as the samples’ loading; (2) reflecting the true response of the structure in rheological tests, and being able to obtain the variability of the dependent variables within the scope of the study; and (3) ensuring that test results have ideal repeatability (this is extremely challenging in particulate dispersions). Although some current work attempts to erase shear history to achieve ideal viscoelasticity and repeatability, it also inevitably wipes out original information. In terms of viscoelasticity, it may be valuable within a certain range, especially under small disturbances, and their nonideal fluid properties cannot be ignored.

As a typical time-dependent suspension, the dynamic structures of particulate gel fuels are frequently sensitive to prior flow history. The initial structure, containing various shear histories, governs the transition from a solid-like state to a liquid-like state [18]. Indeed, particulate gels experience structural breakdown and reformation in response to external forces or shear. The balance between these two processes controls the matrix’s relative elastic and viscous responses. These behaviors are typically time-dependent; prolonged shear allows the structure more time to relax or remodel, leading to thixotropic-like changes in the micro structure. Heymann et al. [19,20] discovered that stress was only correlated with strain or strain rate at low or high shear rates. The results illustrated the prominent features of elasticity and viscosity in the two regions, respectively. During the transition, a minimum value of shear stress emerged, which was related to the loading time and accompanied by a strong nonlinear rheological response [21]. In gel fuel composed of hydrocarbon fuel and FS [15], a stress-relaxation behavior was observed and could be modeled by an extended Herschel–Bulkley model [22]. Those findings imply that shear time significantly influences gel structure.

Similar research indicates that carbon black gels [23] would undergo slow solid–liquid evolution under the prolonged action of oscillatory shear, attributed to the dynamical fatigue scenario of the particulate system. Shakeel et al. [24] also observed that mud sediments took longer to reach equilibrium viscosity under higher shear. This phenomenon may be associated with structural damage and stress relaxation, both of which are significantly influenced by temperature or preshear amplitude [25,26].

Shear-induced phenomena, particularly structural changes within particulate gels, are a significant focus when studying shear history. These changes, often accompanied by variations in particles, are prevalent across various gels. The goal of such studies typically involves understanding and integrating the properties of structures that emerge because of shear. Initially, Pignon [27] emphasized the importance of observation, in the recovery situation, for these nonideal behaviors after the structure was completely destroyed under high shear. Based on the ideas above, Sun [28] and Shu [29] investigated the recovery structure of Laponite suspensions in large amplitude oscillatory shear (LAOS) with a preshear at 200 s^−1^ or even 3000 s^−1^. However, this approach may not be suitable for some actual industrial products if examining their native or compliant flow [30].

While undertaking a concentrated suspension filled with polystyrene spheres, Gadala et al. [31,32] discovered the formation of shear-induced new structures through the evolution of stress or viscosity over time. The subsequent results showed peaks in the distribution function of the suspension in the flow direction, indirectly proving the anisotropic features of the structure. Notably, when the shear direction changed, the distribution of clusters in the network became a mirror structure. In concentrated suspensions of Al particles, Guo et al. [33,34] found that strong shear history (high-speed shear over a long time) would increase the number of particles building the network. Consequently, the system exhibited a trend of structural thickening and solid–liquid transition. The competition and balance between gravity and viscous forces caused the system to exhibit yield stress and shear banding phenomena. Based on previous studies, Choi et al. [13] obtained unbiased flow curves after inserting a strain of 100% in the direction opposing the high shear rate step, explaining that this was related to the elimination of residual stresses under shear. In general, current research has primarily concentrated on qualitative assessments of network structures in gels, often overlooking the variations in structural strength. However, understanding these variations of quantitative characterization is crucial, as they are fundamental to the engineering applications of gels.

Typically, rheological measurements depend on a preparation process and testing protocols. Particulate gels tend to suffer from wall slip, particle migration, or phase separation during shear, which reduces measurement accuracy [35]. This is also what has been overlooked in studies on shear history. Generally, a slotted rotor [36] or parallel plates equipped with a rough surface [37] are adopted to obtain nonslip rheological properties. Owens et al. [25] introduced modified vane fixtures with fractal-like structures in the rheological study of yield stress fluids. They found that vane geometry was effective in eliminating wall slip and disturbing the macroscopic/bulk structure of the sample. Similarly, the vane rotor has become increasingly accepted in the study of agricultural by-products [38], geotechnical fields [39], daily necessities [40], etc.

In fact, no variability in the concentrated suspension could be identified through the variation of the shear direction. For most studies, using a single preshear empirically and trying to eliminate shear history presents potential problems without considering the practical structural changes [41,42]. Even under small shear, provided sufficient time and critical strain are met, few studies have been carried out on whether particulate gels will undergo tensile fracture like solid materials, nor on the recovery of structure after such fractures. Therefore, understanding the structural evolution of gel fuels under different degrees and durations of shear is fundamental and attractive work. For particulate flocculation systems, nonideal phenomena such as shear bands and wall slip haunt rheological testing like “ghosts”, leading to many unreal elements in the results. However, few studies focus on and address this issue. It is well known that a preshear is performed before rheological testing, but this preshear is mostly empirical, and many testers do not even adopt it. Thus, this issue is often avoided, and very few studies face it and attempt to provide answers. Especially in application scenarios, different shearing programs may yield different results, potentially affecting further expansion of applications. Based on the above confusion, the impact of shear history from the most basic scales of magnitude and duration of shear was explored. In this process, different shear rates were used to obtain related structural shear spectra, which helped us better understand the micro-flow mechanisms of particulate structure during shearing and provided evidence of structural destruction and reconstruction.

In this paper, a new observation was used by applying the different shear procedure on the particulate gel fuels using in situ sample loading and a four-arm vane rotor for the first time. In this work, repetitive, direction-changing continuous shear ramp was applied. The time-dependent rheological responses were analyzed using a new protocol consisting of steady shear (${\dot{\gamma }}_{k}$ = 0.1, 1, 5, 10, 50 and 100 s^−1^), recoverable shear (${\dot{\gamma }}_{\mathrm{eq}}$ = 0.001 s^−1^), and a small amplitude oscillatory shear (SAOS, *γ* = 0.1 to 0.5%). The results will provide insight into the flow mechanisms of FS and Al MPs via morphological observation and rheological texts and constitute attempts to reveal the transient breakdown and recovery behaviors of the mechanical behavior of complex gel fuels, providing an expanded understanding of the solid–liquid transition.

## 2. Results and Discussion

### 2.1. Morphological Characteristics

**Figure 1:**
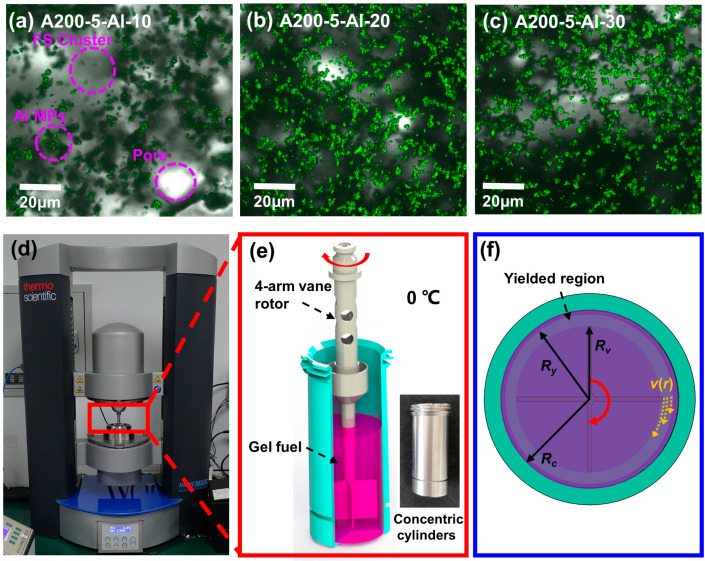
CFM images of the aggregate structure in dispersions formed by FS and Al MPs in a binary fuel mixture of heptane/isopropyl nitrate at different particle concentrations. Confocal fluorescence images of gel fuels with a merged channel of A200-5-Al-10 (**a**), A200-5-Al-20 (**b**), and A200-5-Al-30 (**c**). The MARS 60 apparatus (**d**); schematic of the vane rotor inserted into concentric cylinders of gel fuels (28 mL) for measurement (**e**); cross-sectional view of the test device used to assess the rheological properties of gel fuels (**f**).

The microstructure of the particulate gel fuels formed by FS NPs and Al MPs was observed with CFM. For metallized gel fuels (Figure 1b–d), the Al MPs emitting green fluorescence are uniformly distributed in an aggregated form in the dark field picture. The pores of the network become increasingly visible as the Al MPs content increases. A closer look at the FS cluster and Al MPs reveals a tendency that both types of particles are spatially distributed and overlapping; that is, the surface of the naked particles might adhere to the network at the submicron and micron scale, and a network was formed by flocculation into clusters among FS NPs. Al MPs and their aggregates resist the gravitational sedimentation through the adhesion forces generated by the FS-based clusters. However, similar adhesion has been observed previously by Basrur et al. [43]. The gel fuels no longer follow the Stokes equation or Kynch’s theory of sedimentation. Furthermore, as shown in Figure S2b, the diameter (*D*) of pores in the network can be roughly calculated as approximately 25~900 nm using SAXS (*D* = 2π/*q* [44]). As the filling amount of Al MPs increases, the number of pores increases within the test range, indicating the participation of Al MPs in the network’s construction. Whitby [45] investigated the dispersion characteristics of FS in triglyceride solvent and hypothesized from CFM images that FS mainly relies on the overlapping of chain segments to accomplish flocculation and gelation, whereas the study in this paper focuses on the morphology of dispersion and the presence of Al MPs in the FS-based gel structure, and quantitatively characterized the size of the associated pores.

**Figure 2:**
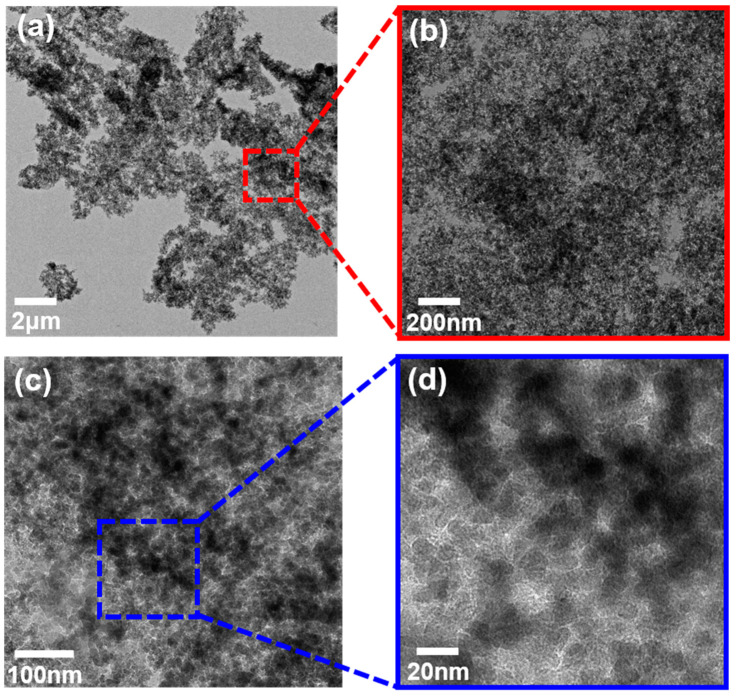
Cryo-TEM image of A200-5: 10,000× (**a**); 100,000× (**b**); 200,000× (**c**); 1,000,000× (**d**).

Considering that FS clusters did not produce visible fluorescence under laser excitation, we tried to characterize the spatial correlation via cryo-TEM. As shown in Figure 2, it can be found that the clusters were heterogeneously distributed on the mesoscopic scale, and the overlapping chains form an isotropic fragile network structure. In addition, the FS-based clusters were amorphous and formed at the micron scale, and it was found that the density in certain areas was different after zooming in, as shown in Figure 2b, which was related to its 3-D structure. In Figure 2c,d, we can identify the particles inside the flocculated cluster. Among them, the primary particles were approximately 14 nm, and the clusters were dispersed into the continuous phase. The surface of the primary particles was connected by hydrogen bonds and existed in a dense distribution [46].

### 2.2. Repetitive Ramp Shear Sweep

**Figure 3:**
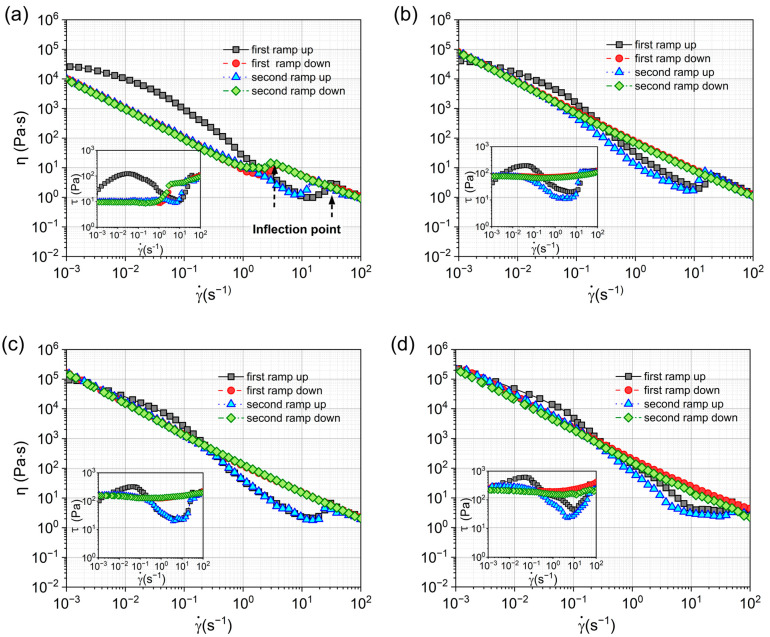
Viscosity and stress curves of (**a**) A200-5, (**b**) A200-5-Al-10, (**c**) A200-5-Al-20, and (**d**) A200-5-Al-30 were measured by the shear procedure: shear rate was increased from 10^−3^ to 10^2^ s^−1^ (first ramp up), varied from 10^2^ to 10^−3^ s^−1^ (first ramp down), increased from 10^−3^ to 10^2^ s^−1^ (second ramp up) and finally decreased from 10^2^ to 10^−3^ s^−1^ (second ramp down).

Gel fuels, as the typical particulate system, require a particular level of driving force, known as yield stress/strain, to begin flowing. To deduce the variance in flow characteristics for both metallized and nonmetallized situations, the samples were exposed to repetitive ramp shear sweep with a shear rate ranging from 10^−3^ to 10^2^ s^−1^, as shown in Figure 3. The test procedure for the ramp shear sweep was similar to the thixotropic loop test, and the sweep was repeated. The shear rate of the first ramp up increased from 10^−3^ s^−1^ to 10^2^ s^−1^, which was recorded as the “first ramp up”; the subsequent shear rate decreased from 100 to 10^−3^ s^−1^, which was recorded as the “first ramp down”. Repeating the above operations, we recorded them as the “second ramp up” and “second ramp down”, respectively. No preshear was used before testing to ensure that the rheological signal was the most original mechanical response.

**Figure 4:**
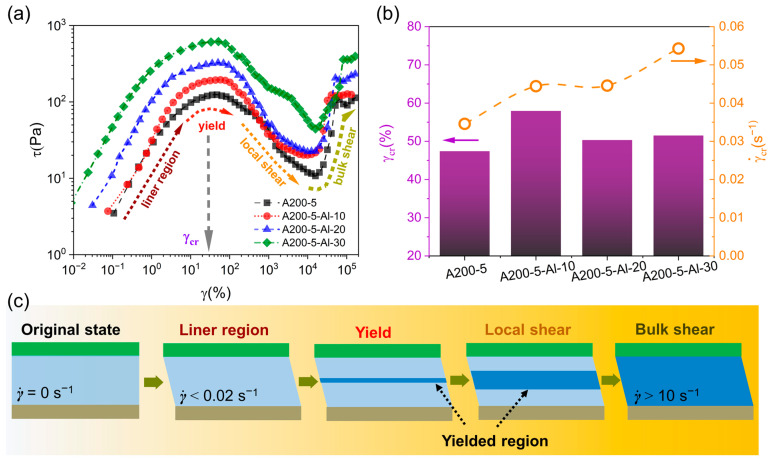
Flow curves of first ramp up for *τ* vs. *γ* (**a**) and the *γ*_cr_, $\dot{\gamma }$ of gel fuels (**b**). Schematic diagram of the shear-region variations (**c**).

However, in order to capture the initial structural information under shear, we firstly analyzed the typical flow curve of the first ramp up. As shown in Figure 4a, the stress of the Matrigel (A200-5) initially increased until it reached the maximum value ($\dot{\gamma }$ < 0.055 s^−1^) within the scope of this study, and then decreased to a minimum value approximately 10 s^−1^. When $\dot{\gamma }$ > 10 s^−1^, all samples exhibited essentially the same tendency of increasing stress and viscosity, a phenomenon highly reminiscent of shear thickening behavior [22,47].

In detail, A200-5 showed a typical linear response with the strain (*γ*) increasing in the range of 0.01 to 50%. The result from the oscillatory stress sweeps (Figure S2c,d) suggests that the linear region below 0.02 s^−1^ can be easily observed. Once $\dot{\gamma }$ exceeded the value of 0.02 s^−1^, the gel fuels began to yield and reached the maximum at critical strain (*γ*_cr_) as shown in Figure 4b. Along with the increase in $\dot{\gamma }$, the gel fuels exhibited a typical shear thinning performance (Figure 3). If the viscosity was fitted by the power law relationship (Equation (1)), the value of *n* can be found to gradually decrease with the increase in $\dot{\gamma }$:

(1) $${\eta =\dot{\gamma }\;}^{n-1}$$

Kawaguchi et al. [48] found that the increase in stress under high shear was accompanied by an increase at first and then a decrease in I(q) at 0.1 nm^−1^ (pore diameter was 63 nm) when tested by SANS. This suggests a potential increase in the number of holes which can be attributed to a reduction in the volume fraction of the hydroxy anchor attachment sites. The FS-based gel system mainly relies on flocculation and gelation formed through hydrogen bonding between hydrophilic silica particles [9]. This is also the essential control mechanism for the breakdown and reconstruction of the gel structure. That is, 3D networks generate more pores between the particles and the mother liquor under high shear. However, the rapid transformation from dense state to sparse state might run contrary to the hypothesis of shear thickening. In fact, the stress growth can be well related with the evolution of the shear layer or yielded region supported by Pignon [27]. From an experimental viewpoint in this study, as shown in Figure 4c, the structural destruction started at a narrow shearing plane that bore almost all deformation. With an increase in the shear, the plane gradually expanded and formed a yielded region.

Above all, the flow curve can be separated into four stages as follows: (1) $\dot{\gamma }$ < 0.02 s^−1^, in which the stress increases linearly with an elastic response corresponding to the linear viscoelasticity (LVE); (2) 0.02 s^−1^ < $\dot{\gamma }$ < 0.05 s^−1^, in which the sample yields and reaches *γ*_cr_ (≈50%) at around 0.05 s^−1^; (3) 0.05 s^−1^ < $\dot{\gamma }$ < 10 s^−1^, in which a rapid drop of stress accompanied by an increase in the yielded region and shear-band propagation; (4) $\dot{\gamma }$ > 10 s^−1^, in which the shear band extends across the entire gap, and the shear is obtained throughout the bulk of the sample with a velocity gradient of $\dot{\gamma }$ (Figure 1f).

In Figure 3, which displays the results of the thixotropic loop test. It can be observed that not all curves adhere to monotonic power-law behavior. Particularly during the first ramp up, the samples exhibited different slopes across four distinct stages. These stages can be individually fitted using Equation (1) to determine the corresponding consistency coefficients and power law exponents. A similar fitting process was applied to the data from the other three ramp processes, with the comprehensive results presented in Table 1. This analysis reveals that each stage can be modeled with a power law equation. Notably, there is a significant decrease in the power law exponent during and especially after yielding, which suggests intense shear-thinning behavior; this implies that the shear and deformation are predominantly concentrated within a narrow band. Furthermore, the addition of Al MPs primarily increases the consistency coefficient, thereby enhancing the Matrigels’ strength.

On the other hand, a distinguishable difference in Figure 3 can be noticed between the first ramp up and the first ramp down. The shear applied to the gel fuels during the first ramp up was high enough to destroy the gels’ structure. The applied shear had an impact on the gels’ structure and rheology. For the Matrigel or aluminum-containing fuels, they did not reach the stress peak that appeared in the first ramp up during the second ramp up process. That is, gel fuels did not regain their original structure via self-repairing ability when the shear was removed during the time scale in this study. It can be hypothesized that this structural damage formed during testing may need to be repaired by stronger dispersion methods, such as ultrasonic dispersion, that provide enough energy input to allow as many inter-particle connections as possible to rebuild the network [15].

In the first or second ramp down (Figure 3), the flow curves approximated straight lines, following the ideal power law, with no decrease observed at around 10 s^−1^. However, for the second ramp up of aluminum-containing gel fuels (A200-5-Al-10, A200-5-Al-20, and A200-5-Al-30), the curves of viscosity and stress at high shear exhibited a good repeatability with the first ramp up. This proves that 3D particulate structures in ramps are similar in dynamic distribution. In fact, the structural destruction and recovery are obvious when analyzed from the perspective of the thixotropic loop test. However, this study revealed that FS gel-based fuels do not exhibit the pronounced thixotropy observed in organic gel fuels, particularly in the region of the thixotropic loop. In other words, particulate gel fuels demonstrate transient structural recovery characteristics, in contrast to the time-dependent thixotropic behavior of organic gel fuels [49].

In general, as shown in Figure 3a, it is worth noting that the inflection points in the ramp down had a certain delay compared to the ramp up. This means, that during the ramp up process, the gel fuels had a lower strength of Matrigel at high shear, which manifested in the lower viscosity and stress required to achieve the same shear rate. However, at higher shear rates, the 3D network is completely broken, resulting in a uniform flow. The dissociation of clusters has a great dependence not only on the shear rate, but also on time. The structure will produce obvious dissociation under long-term shearing, such as dehydration condensation, low strength and other effects, even if the shear rate is small. The reason why we cannot observe the minimum values of stress and viscosity in ramp down is because there is less time (*t*_m_) than relaxation time (*λ*). In a very quick process, the liquid suspension will rapidly evolve to a solid structure with a vanishing transition regime [19]. In addition, similar phenomena were also observed by Snatos [24] and Shakeel [24]; that is difficult for the sample viscosity or stress to reach equilibrium under high shear. Therefore, in this case the gel will be in an unstable transition state, manifested by a sustained decrease in viscosity or stress. This phenomenon will be discussed in the next section.

### 2.3. Structural Evolutions under Different Shear Protocols

To probe the structural evolutions under different shear rates, an experimental protocol was applied for validation as presented in Figure S3 [50]. This protocol consisted of applying consecutive shear procedures with changes in the shear rate and was represented as long shear history (LSH). Every procedure consisted of steady shear (${\dot{\gamma }}_{k}$), recoverable shear (${\dot{\gamma }}_{r}$), and SAOS. The shear rate (${\dot{\gamma }}_{\max}$ < 0.036 s^−1^) in SAOS was low enough that no “shielding effects” would disturb the next procedure. It was supposed that the spectra of the structure at different ${\dot{\gamma }}_{k}$ were represented by the shear stress/shear rate. That the structure had a tendency to break down until it reached the equilibrium stress at the end of the stress-relaxation phase ($\dot{\gamma }$ ≡ ${\dot{\gamma }}_{k}$), which can be regarded as stress relaxation. Once the high shear was removed, the fuel underwent structural recovery for 120 s at a quasi-static state of 0.001 s^−1^, and, finally, the SAOS test was performed to explore the structural strength. In addition, a mild preshear was applied for 300 s at 0.01 s^−1^ prior to each procedure, in order to let the vane rotor contact the sample tightly and form an orientational network consisting of FS clusters and Al MPs. By repeating the procedures at different ${\dot{\gamma }}_{k}$, a range of structures can be obtained.

Firstly, this methodology was developed for assessing the dynamic yield stress in thixotropic fluid. Thixotropy can be defined as a reversible decrease in viscosity over time in response to an external force [51]. The thixotropy information can be acquired directly by comparing the stress ($\tau_{r}$) in the rest shear period of the adjacent procedures. However, steady shear at extremely low shear rates (<0.1 s^−1^) is too harsh to carry out in practical applications. Typically, shear history under a high shear rate may have practical implications.

**Figure 5:**
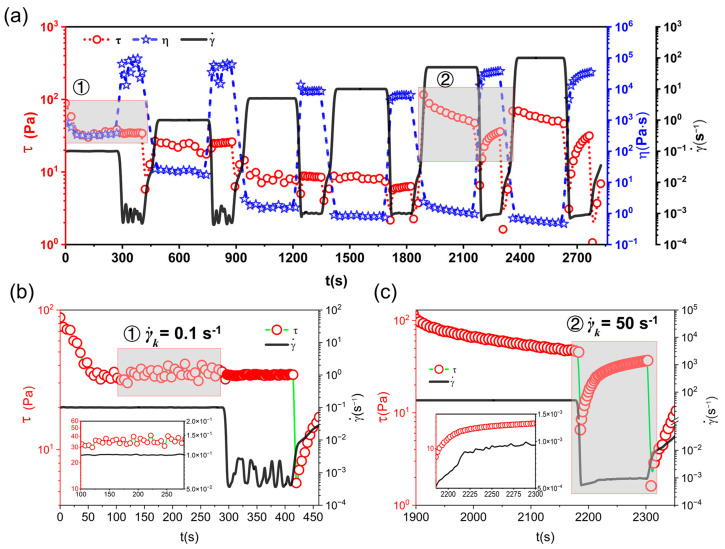
Rheological responses under LSH of A200-5 over the entire observation period (**a**); at ${\dot{\gamma }}_{k}$ = 0.1 s^−1^ (**b**); at ${\dot{\gamma }}_{k}$ = 50 s^−1^ (**c**).

However, in both Figure 3 and Figure 5a, the obvious increase in stress of the Matrigel did not occur when shifted to a higher shear rate (${\dot{\gamma }}_{k}$ ≥ 50 s^−1^). This provides the strongest evidence to argue against the existence of shear thickening under high shear observed by Weston [47] and Kawaguchi [48]. In fact, the particulate suspensions with thixotropy prefer to flocculate [52]; a critical volume fraction of the self-supporting cluster is needed to form the gel. As mentioned in the introduction, the system is highly dependent on the shear history. By applying shear, the gel fuels near the rotor are expected to decompose into a shear-thinning layer and the other clusters continue to be strongly linked. When ${\dot{\gamma }}_{k}$ ≥ 10 s^−1^, the uniformly sheared band gradually extends across the entire gap. As the feedback stress and shear band expand, homogeneous shear can be obtained. In general, when ${\dot{\gamma }}_{k}$ ≤ 10s^−1^, the continuous stress drop indicates that the gel fuels’ structure in the local shear region has gradually been destroyed. When ${\dot{\gamma }}_{k}$ ≥ 10 s^−1^, the increase in the shear region causes the shear stress to increase.

From Figure 5b, we can observe that the Matrigel only took 50s to reach a steady-state structure under a shear of 0.1 s^−1^. In contrast, Figure 5c shows a continuous stress drop from a high stress at 50 s^−1^. This behavior can be attributed to two main factors. Firstly, high shear induces a bulk-shear transition, causing the structure to be continuously damaged, leading to a stress drop. Secondly, the structure is rapidly damaged when subjected to high-speed shear. The observed decrease in stress is associated with the structural recovery and stress relaxation of the gel fuels. This phenomenon is influenced by the Deborah number (the ratio of relaxation to experimental time):

(2) $$De=\lambda /t_{m}$$

when *De* < 1, a fast stress equilibrium can be observed, resulting in a stable gel structure under a constant shear rate. However, as *De* increases (i.e., with an increase in the shear rate), the stress equilibrium becomes unobservable because of the limited experimental time, leading to a consistent decrease in stress [24].

Like the Matrigel (Figures S4–S6), the Al-containing gel fuels exhibited similar variation in stress and were strongly dependent on the applied shear rate (${\dot{\gamma }}_{k}$). The Al MPs reinforced the FS-based Matrigel, which can be connected with the adhesion shown in Figure 1a–c. That is, at the highest volume fraction of particles, A200-5-Al-30 required higher stress to achieve a bulk flow. It is not difficult to determine from our previous research [9] that after aluminum powder is filled into the FS-based Matrigel, the overall rheology depends on the FS, and the role of aluminum powder is only a reinforcing effect. Guo et al. [33] found through research on concentrated suspensions of aluminum that there is a critical volume fraction for the gelation of aluminum powder in the continuous phase of Newtonian fluid. In this system, significant gelation should also occur after the aluminum powder reaches its critical concentration. The gelation effect greatly enhances the strength of the gel.

Furthermore, the stress decline at ${\dot{\gamma }}_{k}$ tended to become smaller with the increase in filler. For example, the ${\dot{\gamma }}_{k}$ near 10 s^−1^ makes it easier for the suspension to exhibit higher stress than low-content samples. When the parameter, $\tau_{k\text{-}eq}$, was appointed as quasi-equilibrium stress by the average stress collected over the last 100 s at ${\dot{\gamma }}_{k}$, then, when ${\dot{\gamma }}_{k}$ = 10 s^−1^, the values of $\tau_{k\text{-}eq}$ were 7 Pa, 15 Pa, 73 Pa and 82 Pa for A200-5, A200-5-Al-10, A200-5-Al-20 and A200-5-Al-30, respectively.

**Figure 6:**
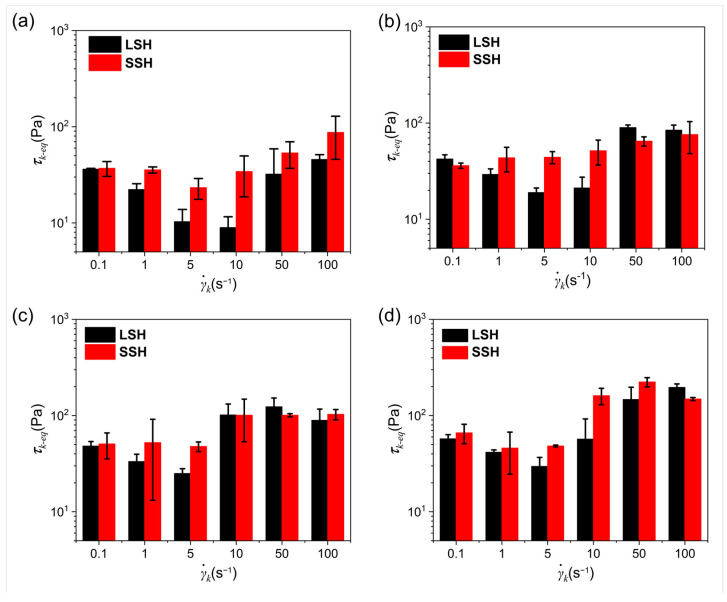
The $\tau_{k\text{-}eq}$ of A200-5 (**a**), A200-5-Al-10 (**b**), A200-5-Al-20 (**c**), and A200-5-Al-30 (**d**).

To explore whether the vulnerable structures were induced by shearing with long durations, a fresh sample was loaded before each shear procedure (Figure S3). These contrast tests were represented as short shear history (SSH). As shown in Figure 6, the values of $\tau_{k\text{-}eq}$ in LSH are lower than in SSH for ${\dot{\gamma }}_{k}$ ranging from 0.1 s^−1^ to 10 s^−1^. As ${\dot{\gamma }}_{k}$ exceeded the threshold (10 s^−1^), especially for the Al-containing gel fuels, no significant differences could be observed between the two models. Similarly, in Figure S7, with the SAOS sweep tracking after recovery shear, we found the G’ of the sample decreased slightly after steady shear. In Figure S7, the G’ of the sample at different ${\dot{\gamma }}_{k}$ in the LSH decreased faster than in the SSH. This means the particulate structure was further ruptured after a long period of shearing. This phenomenon is different from the concentrated suspension system of aluminum particles. Long-term shear would induce Al MPs to participate in building a gel network [33]. In this research system, Al MPs and FS were gelled through strong dispersion methods such as ultrasound before testing. The shear in testing cannot be better than the ultrasonic dispersion effect, so the gel will accumulate deformation under long-term shearing; this causes the gel to break, finally. Once the bulk flow occurred in the samples, the value of G’ also increased within a small magnitude, like the $\tau_{k\text{-}eq}$. However, the variation was progressively suppressed with the increase in the Al MPs. In this study, the gel fuels became increasingly resistant to shear history with the increase in the Al MPs, while the Matrigel was highly sensitive to shear and produced an obvious disruption.

## 3. Conclusions

In this study, a porous FS gel structure was observed, and the flow curves indicated that the particulate gel fuel could be classified into four states: (1) elastic region; (2) yield region; (3) local shear; and (4) bulk shear. After steady-state shear, it was found that prolonged high shear can cause severe structural damage and make it difficult to observe relaxation equilibrium, while the structure equilibrates rapidly under low shear, which may be related to the fact that particle-based gel fuel has a smaller De number under low shear conditions, i.e., the fuel has more time to relax.

## 4. Materials and Methods

### 4.1. Materials

Heptane (AR, ≥99.7%) and isopropyl nitrate (GC, ≥98.0%) was purchased from Aladdin Reagent Co. (Shanghai, China), and the mass ratio of the two components in the primary mixed mother liquor was 4:1. The Al MPs were purchased from Angang Group Aluminium Powder Co., Ltd. (Anshan, China), and they had a particle size distribution in the size range 0.4–4 nm. The hydrophilic FS (A200) was chosen as the gallant for solidifying the mother liquor, with a primary spherical diameter of 12 nm (the BET surface area was 188.2 m^2^/g), which was purchased from Evonik Degussa Co. (Akron, OH, USA). The FS and Al MPs were dried for 24 h under vacuum at 120 °C.

### 4.2. Preparation

The predispersions were prepared by mixing the FS and mother liquor using a homogenizer (Lichen) with an 8 mm blade operating at 30,000 rpm for 10 min. The dispersions were poured into a three-mouth flask, fixed, and swirled while sonicating with a stirring paddle extended within the flask and placed in the ultrasonic disperser’s water bath cavity (900 w, 10 min). Then, the gel matrices were prepared. In the aluminum-based gel fuels, the Al MPs were added to the matrices after mixing the gelatin matrices for 10 min, and the mixing of the aluminum-based gel fuels continued with the use of a mechanical disperser with a paddle rotor. Then, the gel fuels were moved from the beaker to a piping bag and squeezed into a sealed bottle and taken into the ultrasound for 40 min to remove air bubbles and ensure homogeneous quality. The gel matrices and fuels formed a 3D network structure after stirring and sonication and were placed in a sealed bottle. The sealing effect should be strictly controlled to prevent liquid components from spilling over.

### 4.3. Characterization

#### 4.3.1. Confocal Fluorescence Microscopy (CFM)

The microstructure of the gel matrices and fuels were observed using confocal fluorescence microscopy (FV1200, Olympus Corporation; Tokyo, Japan). The Al MPs in the gel fuels were stained with methyl red (50 mg/L in the mother liquor). The staining agent was added dropwise to the mother liquor before the Al MPs were stained. The methyl red was excited at 559 nm and the fluorescence intensity data were collected over the wavelength range of 570–670 nm. The FS clusters would not normally produce any appreciable fluorophore excitation under the above conditions.

#### 4.3.2. Cryonic Transmission Electron Microscopy (Cryo-TEM)

The appearance of the gel network structure was investigated with a Cryo-TEM (Talos F200C, FEI; Hillsboro, OR, USA), which is optimal for observing the in-situ morphology of the Matrigel. Cryo-TEM was employed in the present study to visualize the morphology of the monodisperse FS primary particles and clusters. During the TEM ultramicrotomy, the sample slices with a thickness of 300 nm were cut with an ultramicrotome (Model UC7FC7, Leica, Wetzlar, Germany) under −96 °C.

#### 4.3.3. Small Angle X-ray Scattering (SAXS)

The SAXS measurements were conducted on a Bruker NanoSTAR system and Cu Kα radiation was used as the X-ray source. Determination of the particle size distribution of the Al MPs was carried out using the Malvern Morphologi G3 SE (Malvern Instruments, Malvern, UK).

#### 4.3.4. Rheological Characterization

The rheological parameters were characterized by using a dynamic shear rheometer (Haake Mars 60, Thermo Fisher Scientific; Karlsruhe, Germany) with a 4-arm vane rotor (*R_v_* = 11 mm) and a concentric cylinder (*R_c_* = 13.5 mm), as shown in Figure 1f. Since the gel fuels are sensitive to shear, in order to minimize the error caused by loading, we smashed the end of the sealed bottle when sampling, opened the bottle cap, and allowed it slide to the bottom of the concentric cylinder under the action of gravity. The selected paddle rotor was tested with a falling speed of 50 mm/min and a set gap of 5.3 mm as shown in Figure 1f. All rheological tests were carried out at 0 °C to avoid volatilization. In Figure 1f, inside the vane boundary *r* < *R_v_*, gel fuel ideally moves as a solid part guided by the vane arms, and the radial velocity *v*(*r*) decays from *v* = *ωR_v_* (*ω* is the rotation rate) at the edge of the vane to *v* = 0 at *r* = *R_y_*. In fact, the rotor will generate a dipole stress field from each vane tip, and when it is continuously sheared, a yielded region (shear band) form (*R_v_* < *r* < *R_y_*) [25].

## Figures and Tables

**Table 1:** The fitted values of the power law equation for the samples in the four stages.

Sample	Process	$\dot{\gamma }$ < 0.02 s^−1^			$0.02\;s^{-1}\;<\;\dot{\gamma }$ < 0.05 s^−1^			$0.05\;s^{-1}\;<\;\dot{\gamma }$ < 10 s^−1^			$\dot{\gamma }$ > 10 s^−1^		
		K	n	R^2^	K	n	R^2^	K	n	R^2^	K	n	R^2^
A200-5	first ramp up	610	0.396	0.989	140	0.04	0.997	86	<0.01	0.963	66	0.101	0.992
	first ramp down	15	0.051	0.993	13	0.064	0.993	9	−0.048	0.969	36	0.201	0.927
	second ramp up	12	0.024	0.994	15	0.103	0.993	4	−0.909	0.950	22	0.293	0.991
	second ramp down	15	0.095	0.994	10	0.015	0.993	8	−0.050	0.969	39	0.157	0.931
A200-5-Al-10	first ramp up	1118	0.429	0.991	228	0.05	0.993	77	<0.001	0.890	74	0.132	0.984
	first ramp down	83	0.004	0.995	58	−0.091	0.994	72	−0.034	0.968	72	0.092	0.936
	second ramp up	68	−0.036	0.995	30	−0.239	0.994	15	−0.515	0.979	119	−0.018	0.992
	second ramp down	64	0.095	0.961	67	0.063	0.901	60	−0.054	0.977	55	−0.050	0.997
A200-5-Al-20	first ramp up	1874	0.414	0.961	468	0.118	0.991	140	<0.001	0.906	173	0.039	0.992
	first ramp down	128	−0.044	0.995	116	−0.067	0.994	114	−0.070	0.970	118	0.119	0.933
	second ramp up	146	−0.032	0.995	106	−0.108	0.994	66	−0.297	0.970	31	0.403	0.991
	second ramp down	126	−0.039	0.995	106	−0.081	0.994	126	−0.016	0.967	124	0.006	0.935
A200-5-Al-30	first ramp up	5592	0.464	0.991	1117	0.186	0.985	303	<0.001	0.912	237	0.103	0.996
	first ramp down	221	−0.052	0.994	172	−0.114	0.994	180	−0.098	0.979	169	0.167	0.930
	second ramp up	267	−0.025	0.994	172	−0.137	0.994	106	−0.340	0.971	2	1.071	0.990
	second ramp down	179	−0.032	0.995	168	−0.049	0.993	170	−0.045	0.967	139	0.083	0.936

## Data Availability

Data available on request due to privacy.

## Supplementary Materials

The following supporting information can be downloaded at: https://www.mdpi.com/article/10.3390/gels9110902/s1, Figure S1: The TEM images of the microstructure of A200 (a), (b), the SEM images of Al MPs (c), the particle size distribution of the Al MPs (d); Figure S2: Scheme of preparing Matrigel and aluminum-containing gel fuels for experiments (a), photographs of aluminum containing gel fuels (b), scattering intensity (I) and scattering vector (q) curve via SAXS of the gel fuels (c), stress dependence of moduli (d) and shear rate (e) for gel fuels; Figure S3: The procedure of consecutive shear changed in shear rate to obtain constant structure curve and rheological responses for gel fuels; Figures S4–S6: The rheological responds under applied shear procedure of A200-5-Al-10, A200-5-Al-20, and A200-5-Al-30, respectively; Figure S7: The elastic modulus of A200-5 (a), A200-5-Al-10 (b), A200-5-Al-20 (c) and A200-5-Al-30 (d) under the SAOS test after steady-state shearing and rest shearing.

## References

[1] Chen A., Guan X., Li X., Zhang B., Zhang B., Song J. (2017). Preparation and characterization of metalized JP-10 gel propellants with excellent thixotropic performance. Propellants Explos. Pyrotech..

[2] Glushkov D.O., Nigay A.G., Yanovsky V.A., Yashutina O.S. (2019). Effects of the initial gel fuel temperature on the ignition mechanism and characteristics of oil-filled cryogel droplets in the high-temperature oxidizer medium. Energy Fuel.

[3] Guerieri P.M., Jacob R.J., DeLisio J.B., Rehwoldt M.C., Zachariah M.R. (2018). Stabilized microparticle aggregates of oxygen-containing nanoparticles in kerosene for enhanced droplet combustion. Combust. Flame.

[4] Cao Q., Liao W., Wu W., Feng F. (2019). Combustion characteristics of inorganic kerosene gel droplet with fumed silica as gellant. Exp. Therm. Fluid. Sci..

[5] Thomas J.C., Homan-Cruz G.D., Stahl J.M., Petersen E.L. (2019). The effects of SiO_2_ and TiO_2_ on the two-phase burning behavior of aqueous HAN propellant. Proc. Combust. Inst..

[6] Sabourin J.L., Yetter R.A., Asay B.W., Lloyd J.M., Sanders V.E., Risha G.A., Son S.F. (2009). Effect of nano-aluminum and fumed silica particles on deflagration and detonation of nitromethane. Propellants Explos. Pyrotech..

[7] Wang H., DeLisio B.J., Holdren S., Tao W., Yang Y., Hu J., Zachariah R.M. (2018). Mesoporous silica spheres incorporated aluminum/poly (vinylidene fluoride) for enhanced burning propellants. Adv. Eng. Mater..

[8] Xue K., Cao J., Pan L., Zhang X., Zou J.-J. (2021). Review on design, preparation and performance characterization of gelled fuels for advanced propulsion. Front. Chem. Sci. Eng..

[9] Li J., Ma H., Li Y., Yang Z., He G., Wang B. (2022). The micromorphology and large amplitude oscillatory shear behaviors of hydrocarbon gel fuels filled with fumed silica and aluminium sub-microparticles. Colloids Surf. A Physicochem. Eng. Asp..

[10] Zhang H., Yu K., Cayre O.J., Harbottle D. (2016). Interfacial particle dynamics: One and two step yielding in colloidal glass. Langmuir.

[11] Kamkar M., Sadeghi S., Arjmand M., Sundararaj U. (2019). Structural characterization of CVD custom-synthesized carbon nanotube/polymer nanocomposites in large-amplitude oscillatory shear (LAOS) mode: Effect of dispersion characteristics in confined geometries. Macromolecules.

[12] Goudoulas T.B., Germann N. (2019). Nonlinear rheological behavior of gelatin gels: In situ gels and individual gel layers filled with hard particles. J. Colloid. Interf. Sci..

[13] Choi J., Rogers S.A. (2020). Optimal conditions for pre-shearing thixotropic or aging soft materials. Rheol. Acta.

[14] Wu X., Wang Y., Wang M., Yang W., Xie B., Yang M. (2012). Structure of fumed silica gels in dodecane: Enhanced network by oscillatory shear. Colloid Polym. Sci..

[15] Arnold R., Santos P.H.S., Campanella O.H., Anderson W.E. (2011). Rheological and thermal behavior of gelled hydrocarbon fuels. J. Propul. Power.

[16] Dibble C.J., Kogan M., Solomon M.J. (2006). Structure and dynamics of colloidal depletion gels: Coincidence of transitions and heterogeneity. Phys. Rev. E.

[17] Varga Z., Grenard V., Pecorario S., Taberlet N., Dolique V., Manneville S., Divoux T., McKinley G.H., Swan J.W. (2019). Hydrodynamics control shear-induced pattern formation in attractive suspensions. Proc. Natl. Acad. Sci. USA.

[18] Chen Y., Li X., Zeng G., Liu W. (2015). The influence of continuous shear, shear history and relaxation on the rheological behavior of SiO2/glycerine suspensions. Appl. Rheol..

[19] Heymann L., Aksel N. (2007). Transition pathways between solid and liquid state in suspensions. Phys. Rev. E.

[20] Heymann L., Peukert S., Aksel N. (2002). Investigation of the solid–liquid transition of highly concentrated suspensions in oscillatory amplitude sweeps. J. Rheol..

[21] Hyun K., Wilhelm M., Klein C.O., Cho K.S., Nam J.G., Ahn K.H., Lee S.J., Ewoldt R.H., McKinley G.H. (2011). A review of nonlinear oscillatory shear tests: Analysis and application of large amplitude oscillatory shear (LAOS). Prog. Polym. Sci..

[22] Santos P.H., Carignano M.A., Campanella O. (2017). Effect of shear history on rheology of time-dependent colloidal silica gels. Gels.

[23] Gibaud T., Perge C., Lindström S.B., Taberlet N., Manneville S. (2016). Multiple yielding processes in a colloidal gel under large amplitude oscillatory stress. Soft Matter.

[24] Shakeel A., Kirichek A., Chassagne C. (2020). Effect of pre-shearing on the steady and dynamic rheological properties of mud sediments. Mar. Pet. Geol..

[25] Owens C.E., Hart A.J., McKinley G.H. (2020). Improved rheometry of yield stress fluids using bespoke fractal 3D printed vanes. J. Rheol..

[26] Deboeuf S., Ducloué L., Lenoir N., Ovarlez G. (2022). A mechanism of strain hardening and Bauschinger effect: Shear-history-dependent microstructure of elasto-plastic suspensions. Soft Matter.

[27] Pignon F., Magnin A., Piau J.M. (1996). Thixotropic colloidal suspensions and flow curves with minimum: Identification of flow regimes and rheometric consequences. J. Rheol..

[28] Sun W., Yang Y., Wang T., Liu X., Wang C., Tong Z. (2011). Large amplitude oscillatory shear rheology for nonlinear viscoelasticity in hectorite suspensions containing poly(ethylene glycol). Polymer.

[29] Shu R., Sun W., Liu X., Tong Z. (2015). Temperature dependence of aging kinetics of hectorite clay suspensions. J. Colloid. Interf. Sci..

[30] Ewoldt R.H. (2013). Defining nonlinear rheological material functions for oscillatory shear. J. Rheol..

[31] Gadala-Maria F., Acrivos A. (1980). Shear-induced structure in a concentrated suspension of solid spheres. J. Rheol..

[32] Parsi F., Gadala-Maria F. (1987). Fore-and-aft asymmetry in a concentrated suspension of solid spheres. J. Rheol..

[33] Guo Y., Yu W., Xu Y., Zhou C. (2009). Liquid-to-solid transition of concentrated suspensions under complex transient shear histories. Phys. Rev. E.

[34] Guo Y., Yu W., Xu Y., Zhou C. (2011). Correlations between local flow mechanism and macroscopic rheology in concentrated suspensions under oscillatory shear. Soft Matter.

[35] De Kee D. (2021). Yield stress measurement techniques: A review. Phys. Fluids.

[36] Zhu H., De Kee D. (2008). Double concentric cylinder geometry with slotted rotor to measure the yield stress of complex systems: A numerical study. J. Rheol..

[37] Yang K., Yu W. (2017). Dynamic wall slip behavior of yield stress fluids under large amplitude oscillatory shear. J. Rheol..

[38] Beugre E.Y.-M., Gnagne T. (2022). Vane geometry for measurement of influent rheological behaviour in dry anaerobic digestion. Renew. Sust. Energ. Rev..

[39] Lee H., Kwak J., Choi J., Hwang B., Choi H. (2022). A lab-scale experimental approach to evaluate rheological properties of foam-conditioned soil for EPB shield tunnelling. Tunn. Undergr. Space Technol..

[40] Teoman B., Marron G., Potanin A. (2021). Rheological characterization of flow inception of thixotropic yield stress fluids using vane and T-bar geometries. Rheol. Acta.

[41] Innocenzi P. (2020). Understanding sol-gel transition through a picture. A short tutorial. J. Sol-Gel Sci. Techn.

[42] Wu L., Zhang K., Shi J., Wu F., Zhu X., Dong W., Xie A. (2022). Metal/nitrogen co-doped hollow carbon nanorods derived from self-assembly organic nanostructure for wide bandwidth electromagnetic wave absorption. Compos. Part. B-Eng..

[43] Basrur V.R., Guo J., Wang C., Raghavan S.R. (2013). Synergistic Gelation of Silica Nanoparticles and a Sorbitol-Based Molecular Gelator to Yield Highly-Conductive Free-Standing Gel Electrolytes. ACS Appl. Mater. Interfaces.

[44] Radlinski A.P., Mastalerz M., Hinde A.L., Hainbuchner M., Rauch H., Baron M., Lin J.S., Fan L., Thiyagarajan P. (2004). Application of SAXS and SANS in evaluation of porosity, pore size distribution and surface area of coal. Int. J. Coal Geol..

[45] Whitby C.P., Krebsz M., Booty S.J. (2018). Understanding the role of hydrogen bonding in the aggregation of fumed silica particles in triglyceride solvents. J. Colloid. Interf. Sci..

[46] Raghavan S.R., Walls H.J., Khan S.A. (2000). Rheology of silica dispersions in organic liquids:  New evidence for solvation forces dictated by hydrogen bonding. Langmuir.

[47] Weston J.S., Harwell J.H., Grady B.P. (2017). Rheological characterization of yield stress gels formed via electrostatic heteroaggregation of metal oxide nanoparticles. Soft Matter.

[48] Ando M., Kawaguchi M. (2011). Shear-induced changes in rheological reponses and neutron scattering properties of hydrophobic silica suspensions at low silica concentrations. J. Disper Sci. Technol..

[49] John J., Nandagopalan P., Baek S.W., Miglani A. (2017). Rheology of solid-like ethanol fuel for hybrid rockets: Effect of type and concentration of gellants. Fuel.

[50] Cheng D.C.-H. (1986). Yield stress: A time-dependent property and how to measure it. Rheol. Acta.

[51] Møller P.C.F., Mewis J., Bonn D. (2006). Yield stress and thixotropy: On the difficulty of measuring yield stresses in practice. Soft Matter.

[52] Zheng Z., Song Y., Xu H., Zheng Q. (2015). Thickening of the immobilized polymer layer using trace amount of amine and its role in promoting gelation of colloidal nanocomposites. Macromolecules.

