# Supplementary material for: Effect of Shear History on Solid–Liquid Transition of Particulate Gel Fuels
Source: Gels. 2023 Nov 15;9(11):902. doi: 10.3390/gels9110902 (PMC10671478; doi:10.3390/gels9110902)
Supplement: Supplementary file 1 [file gels-09-00902-s001.zip › gels-2705629-supplementary.pdf]

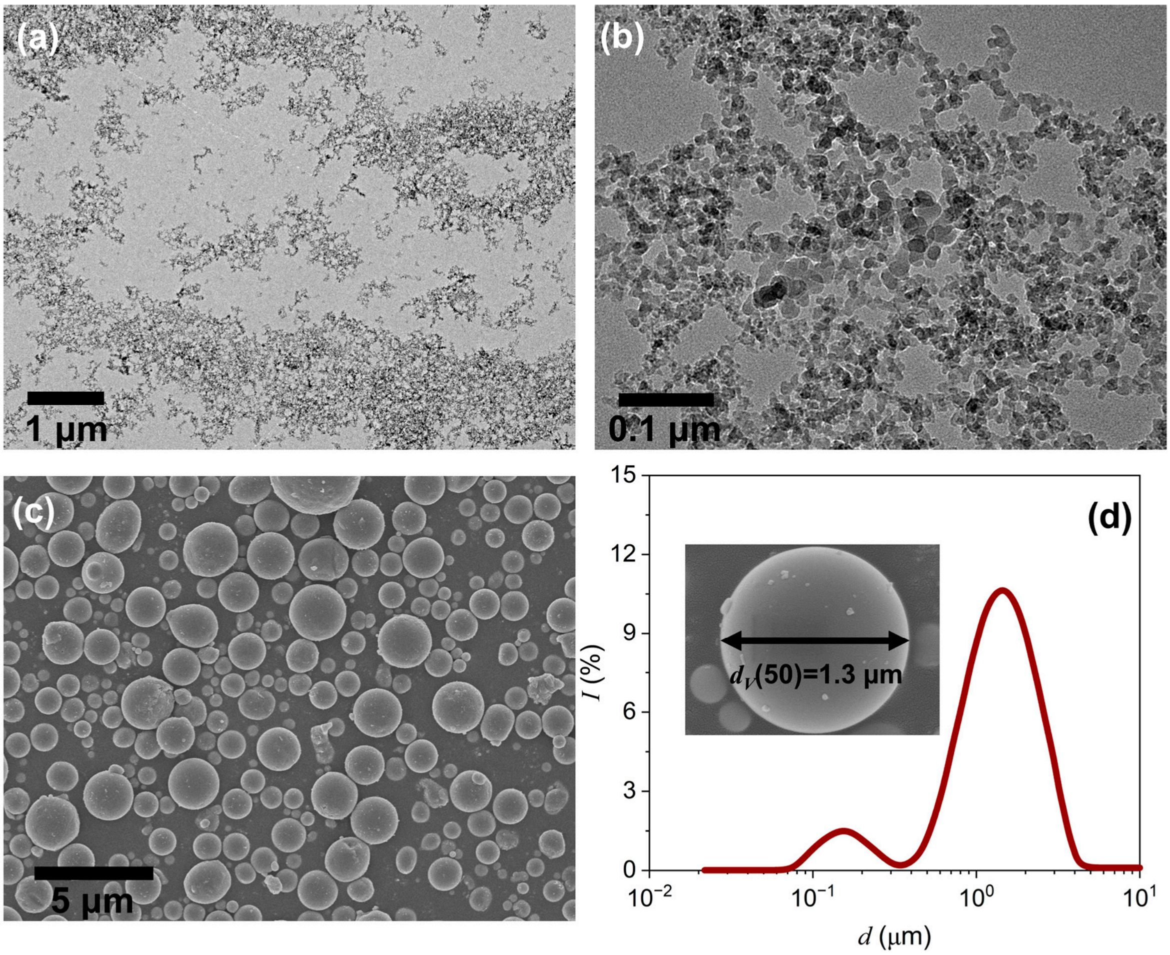


**Figure S1.** Transmission electron microscope (TEM) images of the microstructure in A200 with a primary particle diameter of 14 nm (a), (b). The primary nanoparticles are fused together into clusters with an irregular, chain-like geometry. Scanning electron microscopy (SEM) images of Al MPs with size ranging from 0.1 μm to 3.6 μm (c), the particle size distribution of the Al MPs (d).

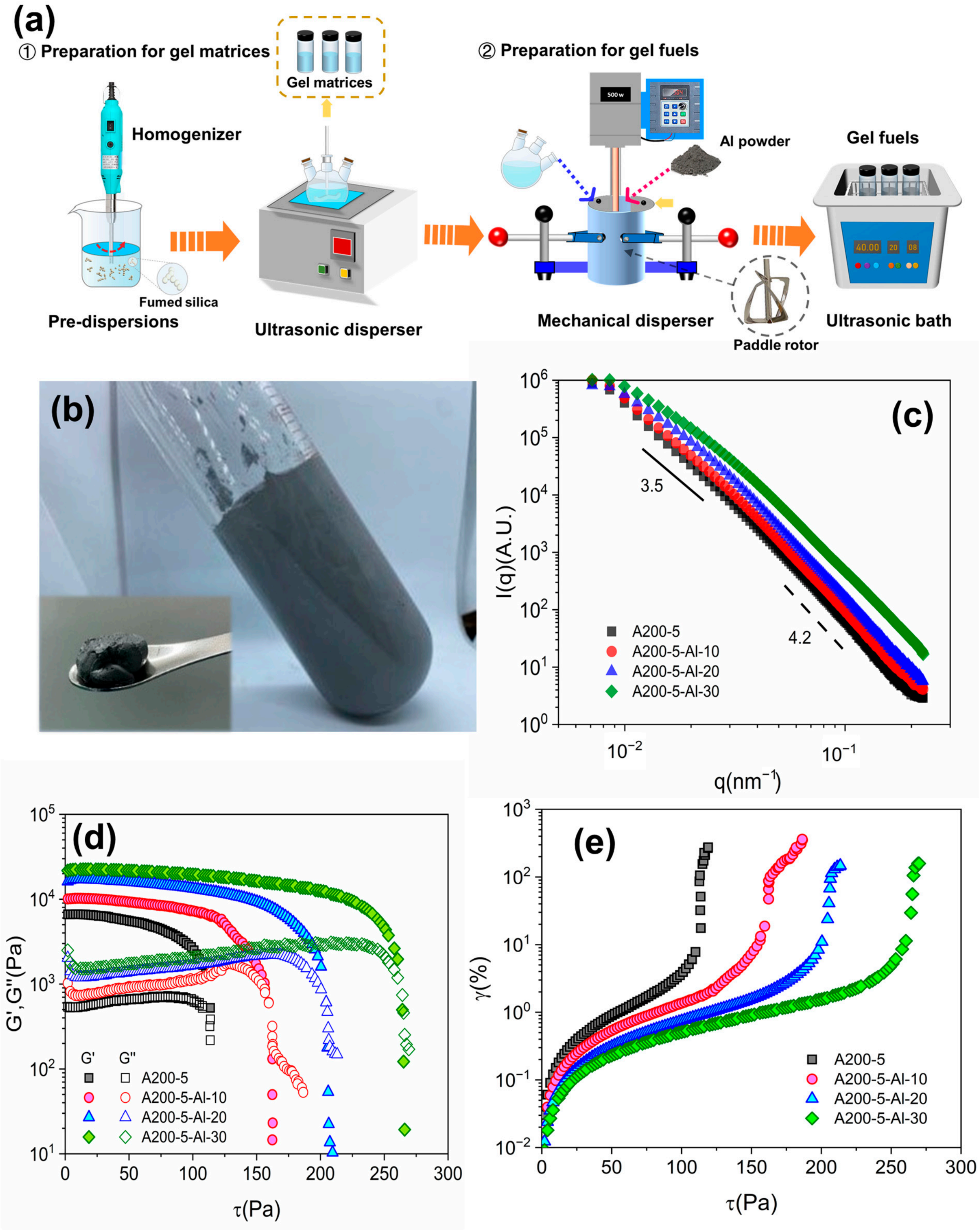


**Figure S2.** Scheme of preparing Matrigel and aluminum-containing gel fuels for experiments (a). Photographs of aluminum containing gel fuels (b). Scattering intensity (I) and scattering vector (q) curve via SAXS of the gel fuels (c). Stress dependence of moduli (d) and shear rate (e) for gel fuels.

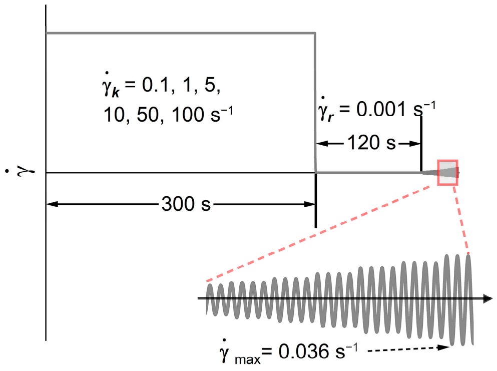


**Figure S3.** The procedure of consecutive shear changed in shear rate to obtain constant structure curve and rheological responses for gel fuels.

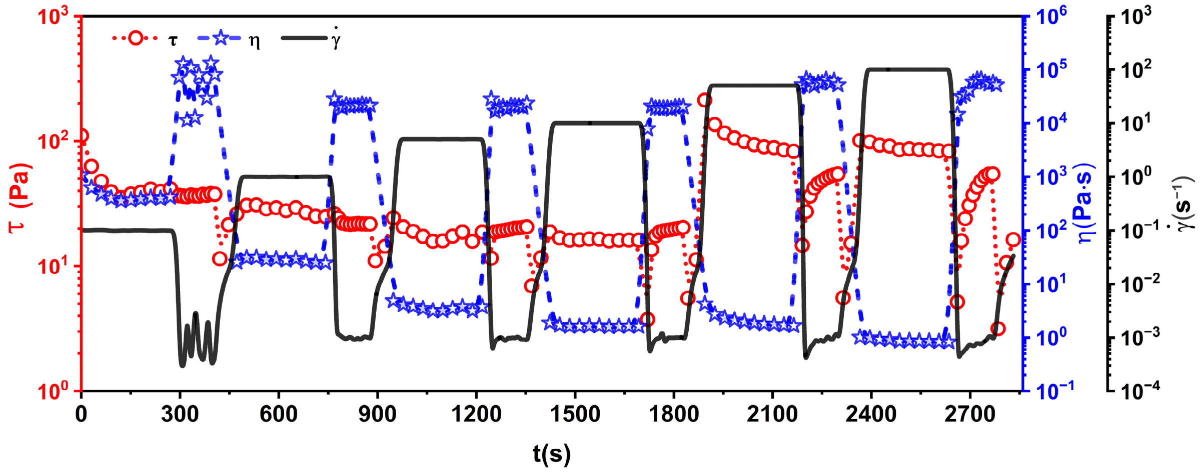


**Figure S4.** The rheological responds under applied shear procedure of A200-5-Al-10.

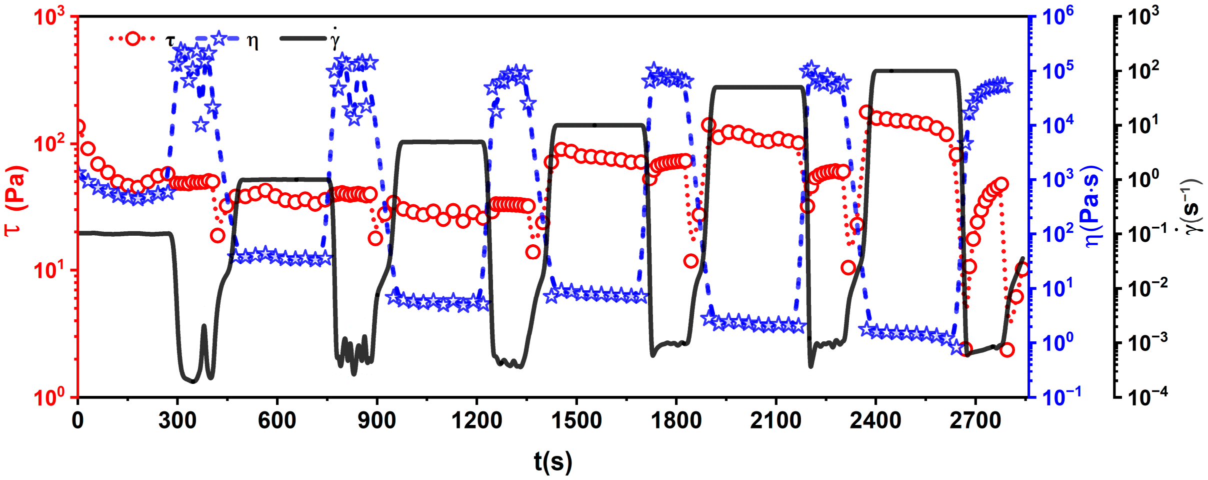


**Figure S5.** The rheological responds under applied shear procedure of A200-5-Al-20.

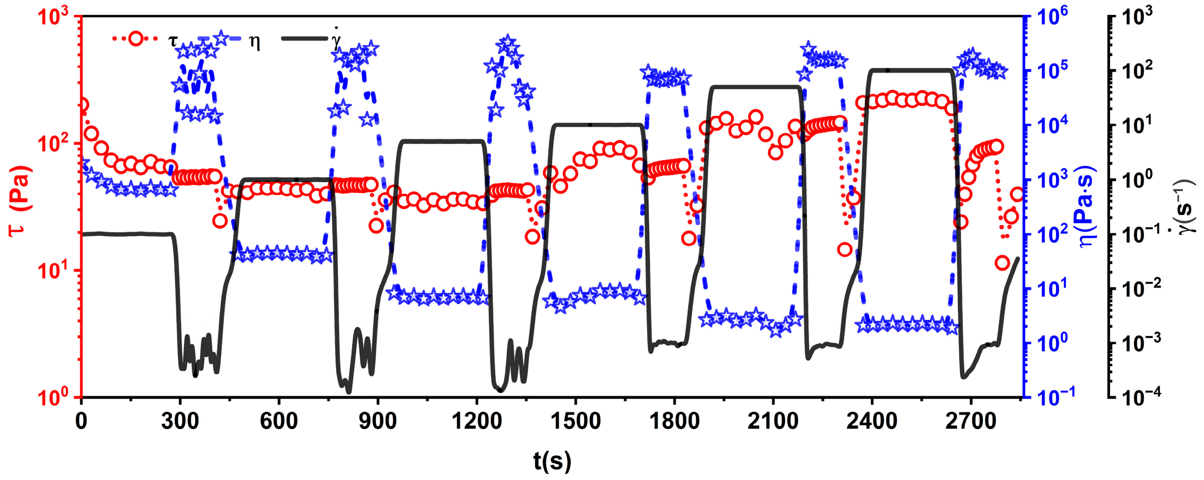


**Figure S6.** The rheological responds under applied shear procedure of A200-5-Al-30.

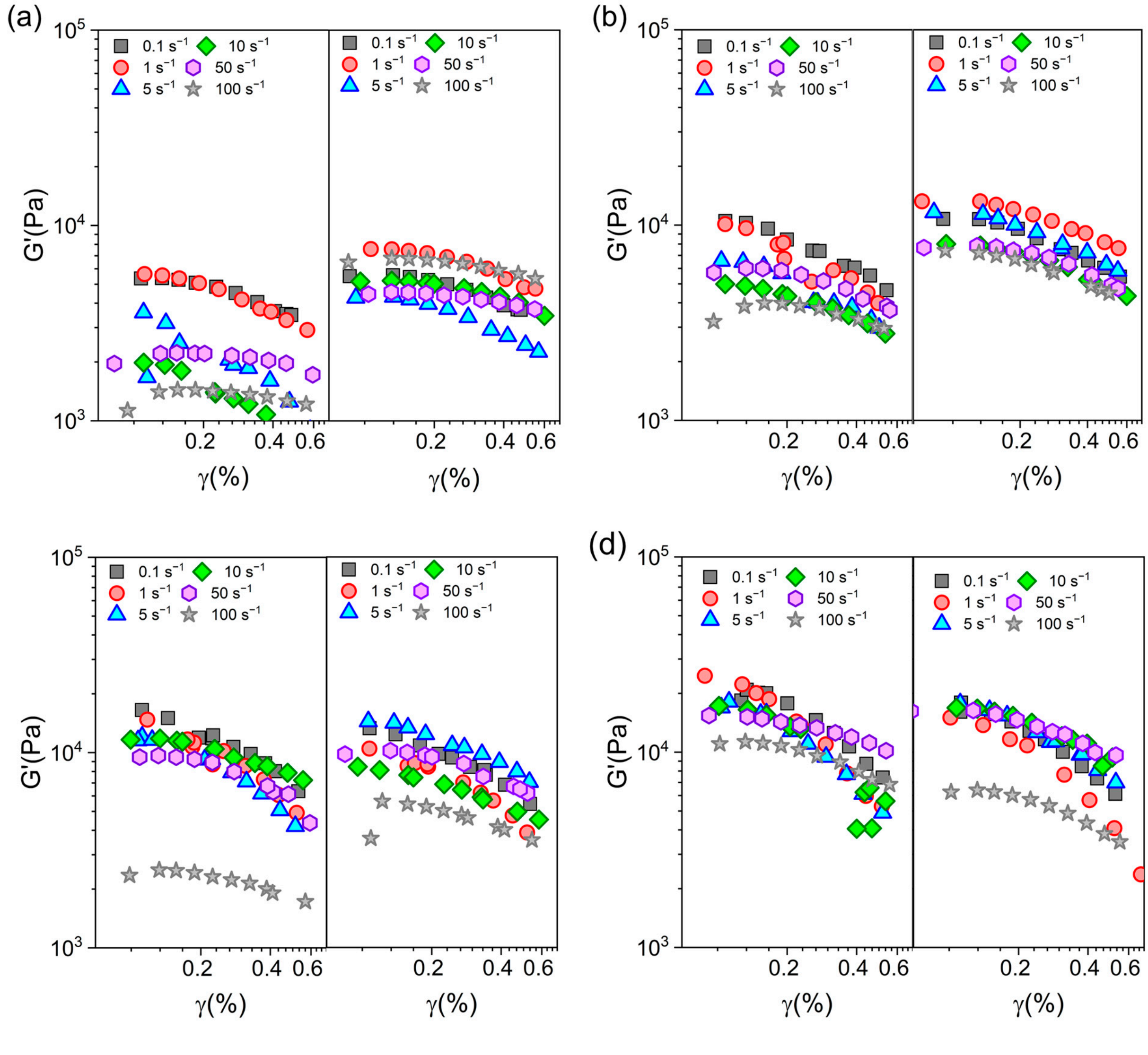


**Figure S7.** The elastic modulus of A200-5 (a), A200-5-Al-10 (b), A200-5-Al-20 (c) and A200-5-Al-30 (d) under the SAOS test after steady-state shearing ($\dot{\gamma}$ = 0.1 ~ 100 s$^{-1}$) and rest shearing (0.001s$^{-1}$) for 300s and 120s, respectively.
